# Treatment of Erythematous Lupus &c.

**Published:** 1886-12

**Authors:** 


### Treatment of Erythematous Lupus by the Application of a Mixture of Vinegar and Yellow of an Egg.

Dr. Brocq (*Jour. Med. de Paris*), calls attention to a practical, convenient, and cheap means of treating erythematous lupus, which the author declares is not inferior in efficacy to any other therapeutic method employed in this disease. Equal parts of the yellow of a fresh egg and ordinary vinegar well beaten up together, and allowed to macerate for twenty-four hours, may be applied in two or more layers over the affected surface. Or a paste made of the yellow of a hard boiled egg, triturated with vinegar, macerated for several hours, and spread upon a piece of flannel, is applied every night, care being taken that it shall extend beyond the borders of the diseased patch, and the next morning it is to be washed off with black soap.

This procedure does not occasion pain, causes only a slight inflammation, is well tolerated by patients, and is followed by a notable improvement. In addition, it does not demand, like scarification or the application of pyroligenous and pyrogalic acids, the surveillance and constant intervention of the physicians.

